# Barriers and facilitators for using research-based knowledge - A qualitative study on first-line managers’ perspectives in municipal health and care services

**DOI:** 10.1186/s12913-026-14666-0

**Published:** 2026-05-07

**Authors:** Maria Bjerk, Cille Hagland Sevild, Oddvar Førland, Lars Jørun Langøien

**Affiliations:** 1 Norwegian Institute of Public Health, Oslo, Norway; 2 Stavanger Municipality, Stavanger, Norway; 3 Western Norway University of Applied Sciences, Bergen, Norway

**Keywords:** Evidence-based practice, Research-based knowledge, Knowledge translation, Municipalities, Health care, Management, Leadership

## Abstract

**Background:**

Research-based knowledge (RBK), together with the users’ values and preferences and employees’ experiences and skills, forms the essential foundation for evidence-based practice in health services. In Norway, as well as in many other countries, local municipalities are comprehensive deliverers of health and care services. Nevertheless, they face major challenges related to accessing and integrating new research into their services. First-line managers in municipal health care are crucial in creating an organisational culture to facilitate the application of RBK. This study aims to explore barriers and facilitators for using RBK in municipal health and care based on first-line managers’ experiences.

**Methods:**

We conducted individual semi-structured qualitative interviews with twelve first-line managers in municipal health care services. We did a framework analysis to explore their experiences and perspectives.

**Findings:**

Four core themes were identified from the analysis: 1) organising for RBK, with the subthemes; current barriers for employing RBK and visioning the ideal promotion of RBK, 2) culture for RBK, with the subthemes; the inner culture and culture shaped by external conditions and expectations, 3) motivation for RBK, with the subthemes; first-line managers perspectives on their own motivation for RBK and on their employees´ motivation for RBK , and 4) barriers and facilitators related to competence on RBK, which we created as an overarching theme, with competence on the individual level and the system level as subthemes.

**Discussion:**

The managers experienced a gap between what is expected and what they can do. On a national or regional level, gathering research-based information and having supporting networks could facilitate the application of RBK. Locally, ensuring RBK in strategic documents, planning competence for RBK, emphasising RBK activities and having professional development roles supporting the managers were found to be important.

**Conclusion:**

First-line managers experience several barriers and facilitators in applying RBK in clinical practice related to organisational, cultural, motivational and competence elements in municipal health care. Both on the local and national level efforts are needed to support the integration of RBK into clinical expertise and the users' experiences, thereby strengthen municipal health and care services.

**Supplementary Information:**

The online version contains supplementary material available at 10.1186/s12913-026-14666-0.

## Background

Research-based knowledge (RBK), together with the users’ values and preferences and employees’ experiences and skills, forms the essential foundation for evidence-based practice in health services [1, 2]. This interaction is particularly challenging to achieve in a municipal context. Compared to other settings, as specialist care, municipal health and care services has a less developed research basis for practice and governance, despite their complexity and scope [3, 4]. Diverse and long-term user needs, combined with limited resources and a lack of tailored, actionable research, create major barriers to applying evidence-based knowledge in local contexts [5–8].

Local municipalities are the lowest administrative units in most welfare states. Nevertheless, in many western countries, including Norway, it is a comprehensive system in which institutions such as kindergartens, daycare centres, primary schools, and health and care facilities are organised [9]. In Norway, municipal health care covers the locally organised health and care services that municipalities are legally obliged to provide under the Health and Care Services Act. This includes general practitioners and out-of-hours primary care, health centres, school health services, physiotherapy and rehabilitation, home-based nursing and care, and municipal institutions such as nursing homes. National authorities define economic, legal and professional frameworks for the services; municipalities plan and operate these facilities. Health and care services are now the largest single sector under municipal authority and new tasks are constantly being allocated [10]. Since the “Coordination reform” in 2012, municipalities have been given responsibility for patients with increasing health complexities, including multiple chronic conditions, frailty and dementia, complex rehabilitation needs after hospital stays, severe mental illness with substance use, and advanced palliative care at home [11]. The Norwegian municipal accounts for 2023 showed that municipalities spent an average of 40 % of their net operating budget on health and care sector [10].

Municipalities play a crucial role in implementing and embedding measures for health and care service quality, for promoting health and achieving health equity. First-line managers or mid-level managers in municipal health and care services are responsible for both personnel and professional quality and are consequently crucial in creating a culture to facilitate the application of RBK in their units. Several studies have shown the central role of managers in these processes [12–14]. To support the Norwegian municipalities, there are several national and regional centres providing competence support within specific topics, for instance, ageing or addictions. However, this support varies between the centres and regions [15]. From 2020, “The Municipalities’ Collaborative Arena for Research (KSF)” has been developed as a collaborative structure that aims to strengthen the basis for research-based decisions and service development in the municipal sector [16].

Internationally, systematic reviews confirm persistent barriers to research utilisation in primary and municipal health care, including lack of access to relevant studies, insufficient time for practitioners, and a mismatch between academic outputs and practical needs [17, 18]. These findings underscore the need for collaboration between researchers and practitioners to ensure that the research that is produced is applicable and relevant to the challenges faced by municipal health and care services [19, 20]. Previous research within implementation science highlights that implementing RBK in health and care services depends on multiple factors beyond the quality of the evidence. Organisational context, leadership, resource availability, and staff motivation all play critical roles in whether evidence is successfully translated into practice [21–24]. In the Nordic context, a few studies have investigated conditions that promote and hinder the use of RBK within the municipal health and care services. These studies have shown that supportive managers, workplace culture and professional commitment and competence are of great importance when implementing evidence-based interventions [25–27]. However, little is known about how these factors interact within the different municipal health and care settings and across the professionals they employ, creating a gap in understanding practical strategies for fostering evidence use at the local level.

Evidence-based practice is described as the employees’ practice based on an integration of evidence from (a) research literature, (b) clinical state, circumstances and assessments, and (c) patients’ values, preferences and experiences [28]. In this study, we are focusing on the use of RBK in the frame of evidence-based practice. There is a gap in research on how RBK is applied in municipal health and care services, and which factors promote and/or hinder its use. This gap can be studied in different ways and by different approaches. Since first-line managers in Norway are responsible for both the administrative and professional aspects of their departments, we chose to interview this group about their perceptions of and experiences with their department’s application of RBK in health and care service delivery. The research question underpinning the study was: What are the barriers and facilitators for the use of RBK in municipal health and care services, according to first-line managers’ perspectives?

### Theoretical perspective on knowledge and evidence

When we interviewed first-line managers in the municipalities about their experiences with the application of RBK, it was not based in the concept of a knowledge hierarchy where research is more important for practice than employees’ experience-based knowledge and expertise and the users’ experiences, values, and preferences. Our point of view is that research-based knowledge is valuable and necessary for knowledge- and evidence-based practice (i.e. when appropriate research exists) *and* that employees’ experience-based knowledge and expertise *and* the users’ experiences, values, and preferences are different and complementary forms of evidence. Thus, the three dimensions of evidence inform each other.

The terms evidence-based practice and knowledge-based practice are often used synonymously as practices based on science and research. We find it necessary to clarify our understanding of the phenomena and concepts of knowledge and evidence. In this paper, we adopt Aristotle’s distinction between three forms of knowledge [29]: episteme, referring to universal and theoretical knowledge (“know that” and “know why”); techne, practical and experience-based skills for performing purposeful actions; and phronesis, the ability to exercise good judgment in specific situations. While episteme and techne are essential, they are insufficient on their own; professional practice also requires phronesis, which develops through experience and fosters wise, context-sensitive action [30]. These forms represent complementary aspects of knowledge rather than alternatives [31].

The concept of evidence is multifaceted and varies across disciplines. Nonetheless, common themes emerge in its descriptions, emphasising its role in supporting or refuting a proposition or belief. The word evidence derives from the Latin, “evidentia”, which means clarity, the obvious, the convincing [32, 33]. External evidence refers to generalisable scientific knowledge from research such as literature reviews and guidelines while internal evidence encompasses local data, clinical expertise and patient preferences derived from practice settings [34]. Their relationship is synergistic and a broad and integrated approach to the understanding of evidence is needed [35, 36]. What is evident in practical settings is what is judged and appears certain, correct and appropriate according to both science, clinical consensus and expertise and among patients [37, 38].

To summarize, in a professional context, the use of general research knowledge is necessary when such knowledge is available. However, research knowledge alone is not sufficient. It must be adapted and situated in practical, artisanal and wise professional practices in encountering the user’s situation and experiential knowledge. Evidence-based practice must be understood broadly as practices based on systematically obtained RBK, employees’ experience-based knowledge and expertise, and the users’ experiences, values and preferences [1, 2]. These appearances of knowledge complement each other in decisions and practices.

## Methods

### Study design

We carried out a qualitative study through semi-structured interviews. We then used a framework analysis to explore experiences and perspectives of first-line managers in municipal health and care services. We chose to conduct semi-structured individual interviews to investigate the depth of first-line managers’ experiences and perspectives, as well as to capture the variation that exists between managers. We used the Standards for Reporting Qualitative Research [39] as a guide in the preparation phase to ensure the rigor of our work. In writing this article the Consolidated criteria for reporting qualitative research (COREQ) checklist [40] was applied.

### Sample and setting

Recruitment for the study was carried out from March to May 2024. To identify possible participants, we contacted advisors in The Norwegian Association of Local and Regional Authorities (KS) and the Centre for Development of Institutional and Home Care Services (USHT). We used a convenience sampling strategy where the advisors from our collaborating partners (KS, USHT) contacted possible participants by email and provided them with information about the study. The first-line managers interested in participating in the study emailed the researchers directly. To achieve a broader sample, we asked the advisors to target recruitment to both urban and rural municipalities of different sizes, in different regions in Norway. Fifty-one first-line managers from different municipal health and care services were contacted by the advisors. Twelve agreed to participate. We do not have information of why 39 managers declined participation as the advisors were blinded to the final participants.

### Data collection

We conducted all interviews digitally on a computer in the period April to June 2024. Interviews were recorded using Nettskjema-Dictaphone, a digital platform for safely storing research data. The twelve semi-structured interviews were conducted individually and applied a pre-planned interview guide with certain themes and proposed questions [41]. We developed and used an interview guide including themes and questions concerning existing and needed competence on RBK and on the application of this knowledge in service development (see Appendix 1). The themes and questions were developed based on experiences from one of our previous studies [42]. The interviews were conducted by two researchers where one had the interviewer role while the other had an observer role, adding questions where it was seen fit and taking notes of arising thoughts. MB and OF carried out half of the interviews. CS and LJL carried out the other half. OF, CS and LJL had significant training in carrying out interviews while MB was less experienced. All interviewers are researchers working in the field of health and social care, and have professional backgrounds within nursing, physiotherapy, sociology and anthropology.

### Data management and analysis

Following the automatic transcription in Nettskjema, two authors (MB and LJL) checked, corrected and anonymised the data and ensured that personal information which could lead to the identification of the participants was deleted. Anonymised transcripts were then stored in a folder only accessible by the researchers. We analysed the material using a framework analysis as described by Smith and Firth [43]. All four authors read through the interview transcripts, taking notes on both perceived patterns and what stood out as surprising and important. Afterwards, we collectively discussed impressions and thoughts of the material and created preliminary semantic codes. We then revisited the material individually with these ideas at hand and developed the codes further. All four authors met again and started mapping and drawing together material and developed four core themes which we believed reflected the perspectives of the managers on the barriers and facilitators of the application of RBK in municipal health care. CS and MB led the subsequent stages of analysis by rereading the transcripts, integrating codes, and developing preliminary subthemes. OF and LJL reviewed these proposed subthemes and provided feedback, which CS and MB incorporated. CS and MB then refined and finalised the core themes and subthemes while continuously revisiting the transcripts and identifying illustrative quotations. Table 1 presents an example of this analytic process, including one core theme, its subthemes, associated codes, and sample quotations. All analyses were carried out manually in Word without the use of qualitative analysis software.

**Table 1:** Example of one of the core themes, two subthemes, codes and quotes

Core theme	Subthemes	Codes	Quote
Organising for research-based knowledge	Facilitators and barriers today	Use and prioritization – finances, personnel according to operational requirements Few common meeting points for clinicians when working shifts	“What prevents further education or research from being used is that we are quite old-fashioned organised, really. We have an old-fashioned running rotation of eight weeks, a spinner which goes the whole year, work every-other weekend, and such and such shift length, not meeting time in shifts, not educational days in shifts, and think about that, at the organisational level, I have not facilitated that we can work with topics, talk about topics, look at the broader picture” (ID2)
	Visioning the ideal	Strategy and planning To make the best use of professional resources	“At my management level and the director-manager level in the municipality, we must become better at making it clear that we expect our managers further down the system to make use of that expertise. We must work strategically with it at all levels of the organisation” (ID 6)

### Ethical considerations

The study protocol was developed in close dialogue with the Research Administrative Support Unit at NIPH. We submitted an electronic protocol detailing the project and privacy measures, which was approved by the NIPH review board (ref. 4412–4412). All participants provided written informed consent before their interviews, and could withdraw from the study at any time.

## Results

We interviewed twelve first-line managers from six municipalities of varying sizes. Characteristics of the participants and municipalities are presented in Table 2. The smallest municipality had approximately 6,000 inhabitants, while the largest had more than 700,000. Half of the municipalities are characterised as mid-sized (5000- 19,999 inhabitants), while the other half were large (more than 19,999 inhabitants). These first-line managers worked in municipal health and care services, and had responsibility for, for example, home care services, nursing homes, services to people with disabilities and physiotherapy and occupational therapy. They were responsible for both administrative and professional tasks and for practice development. The size of their departments varied from 6 to 500 employees. The managers had been in their current position between two and 18 years, but most had been there for between two and four years. Some had been in other managerial positions before their current role. Most of the managers had higher education within management. They had different clinical backgrounds as nurses, physiotherapists, medical practitioners, and social educators.

**Table 2:** Characteristics of the setting and participants

Municipality	Manager role	Services	Number of employees	Size municipality
A	Department	Nursing home, home care services, rehabilitation services	160	Mid-sized
A	Department	Nursing home, home care services, rehabilitation services	30	Mid-sized
A	Department	Home care	6	Mid-sized
B	Department	Nursing home	No info	Mid-sized
B	Department	Services for people with developmental disabilities	130	Mid-sized
C	Department	Municipal emergency medical service	30–40	Large
C	Unit	Environmental health care	No info	Large
C	Department	Community medicine	300	Large
D	Department	Home care	500	Large
E	Department	Home care	100	Mid-sized
E	Department	Habilitation	100	Mid-sized
F	Unit	Development of health services	25	Large

Each interview lasted 50 to 60 min and we ended up with 176 transcribed pages for the analysis. In the analysis process we developed four core themes and two subthemes for each core theme connected to barriers and facilitators for the use of RBK. The core themes and subthemes developed in the analysis are presented in Table 3 below. They form the structure for the findings section.

**Table 3:** Core themes and subthemes regarding facilitators and barriers for using research-based knowledge in municipality health and care services

Core themes	Subthemes
Organisational conditions	Current barriers for employing RBK
	Visioning the ideal promotion of RBK
Cultural conditions	The inner culture
	Culture shaped by external conditions and expectations
Motivational conditions	First-line managers’ perspectives on their own motivation for RBK
	First-line managers’ perspectives on their employees’ motivation for RBK
Competence-related conditions	Individual competence
	System for competence enhancement

### Organisational conditions

#### Current barriers for employing RBK

According to the managers, the organisation of municipal health care, including tasks and structure, were influencing the use of RBK. One of the organisational barriers seemed to be a strong focus and pressure on daily operations and ensuring that all clinical tasks were carried out. The managers spent a lot of their time ensuring staff capacity, both having enough staff to carry out the daily tasks related to the users or patients and on recruiting new staff due to high rates of turnover. According to the first-line managers, time and resources for developing services through gathering RBK were often neglected due to the pressure of daily operations. When we asked the managers about the importance of using resources to search for RBK a manager explained:

*We have to be so resource-efficient all the time in terms of finances and time*,* that you don’t have time to work very purposefully on things where you actually see that we should have done something about*,* such as long-term competence work … I definitely should have spent a lot more time on that* ID1

Furthermore, how the health services were structured appeared to be another barrier that hindered the use of RBK. Most healthcare providers worked in shifts and in different teams. In addition, there were many working in temporary positions. So, creating common spaces for discussion and development of services was challenging. It demanded extra effort by the managers to make room in the schedule for the health care providers, for instance, to be able to participate in educational meetings or seminars discussing and applying research literature. One of the managers said:

*What prevents further education or research from being used is that we are organised in an old-fashioned way*,* really. We have an old-fashioned running rotation of eight weeks*,* a spinner which goes the whole year*,* work every other weekend*,* and such and such shift length*,* no meeting time in shifts*,* no educational days in shifts*,* and think about that*,* at the organisational level*,* I have not facilitated that we can work with topics*,* talk about topics*,* look at the broader picture.* ID2

Due to how the services were structured and the high pressure on daily operations, the managers pointed out that they lacked resources, concerning both time and personnel, to use research evidence to develop practice. They also mentioned the lack of support and guidance to apply RBK in their daily work. Several mentioned the importance of professional positions which held the main responsibility for the development of services, for instance professional developer nurses. Moreover, many of the managers also highlighted a lack of strategic planning regarding competence improvement and how they had started on a competence plan which included competence in utilising RBK.

#### Visioning the ideal promotion of RBK

Even though the managers pointed out many organisational barriers of using RBK to develop clinical practice, there were also several facilitating factors. One role that they highlighted as an important facilitator was the professional development role within the unit. They could provide guidance and competence to the other employees and take the lead in development projects. Having a combined position where the employee works both clinically and with professional development was also seen as a successful. One manager said:

*A professional development nurse who then works a little combined*,* both administratively*,* but also out in operation*,* means that you can also see where the shoe pinches. I think it worked very*,* very well that year.* ID5

Other managers emphasised the value of having a dedicated unit or center whose core responsibility was to support the use of RBK in practice, staffed by employees with higher-level academic competence. These units typically served multiple departments—and in some cases an entire region—rather than a single municipality. When managers encountered clinical challenges or questions, they could consult the unit for guidance.

*I think that we have a tradition in our municipality of working with change work*,* to work with improvement work. I am very lucky to have my own unit called Research*,* Innovation and Development. There are two with a completed doctorate*,* and one who is in education*,* and so*,* I don’t remember the number*,* but quite a few with a master’s degree.* ID2

Having access to a development unit would not necessarily solve all their challenges or be considered feasible in smaller municipalities. One manager highlighted that organising future collaboration between municipalities on applying RBK into decision-making would be increasingly important to solve the complex challenges faced in health care. Often, municipalities have similar challenges and could benefit from learning from each other. One manager expressed it like this:

*And there’s something about talking to your neighbour all the time. Other municipalities often have some examples that we don’t have yet*,* but suddenly we’re there too. There is something about the fact that we could get more knowledge more naturally.* ID10

### Cultural conditions

#### The inner culture

One core theme we developed was cultural conditions including how the working culture in the department – as the collective attitudes – varied. There were descriptions of ‘set’ cultures that were challenging to alter, built on attitudes like ‘we do things like we have always done them’. In these cases, it was not regarded as positive to initiate changes. The culture was influenced by “the law of Jante” (meaning a set of social codes in Norway that prioritise collective well-being over individual achievement) which made the potential gains from employees with high academic competence and valuable experience less prolific as new personnel adapted to the existing culture. The knowledge derived from experience, as opposed to knowledge from research, was in some departments considered of higher value, and this could be a barrier to changes being implemented.

*My experience is that there is very*,* very little use*,* and there is very little demand too… But I think it will come with the younger generation because there is a lot more of it in education. So I think it will force its way forward.* ID5

It was common for managers to express that there was a weak culture around the use of research and research-based literature in their departments. Such research literature was not very sought after or used in the units our informants represented. The few who worked with research literature did so in their leisure time because there was no time or culture to do so during working hours:

*I would say that it is little used. I would think that maybe two or three of us are interested in reading research in our free time. […]Out of 160 full-time employees*,* that is*,* 240 employees*,* there are two or three of us who read and share.* ID1

The managers had different views and thoughts on how to approach building a culture where the use of RBK was common and familiar. In some departments, there was an active effort being made towards breaking down the barriers to RBK, by raising awareness of where research knowledge could be found, and to strive towards the strategic goal of research-based services:

*Being able to base things on research-based knowledge is extremely important. And I feel that when we talk about it*,* there are reflection groups linked to these things*,* it gives the employees a sense of security.* ID6

#### Culture shaped by external conditions and expectations

When we asked for explanations for the weak culture of reading and using research, different contextual factors were highlighted, such as variations in financial conditions, staffing and employees’ formal competence, but also traditions and departmental culture:

*I think it’s very much about time*,* finances*,* but also culture. I think we need more people with higher education who see the value of it - and can transform knowledge into practice - differently than what personnel with lower education can do.* ID1

For our informants, a barrier for RBK was how the culture is formed under the pressure of external demands. The inner culture for RBK seemed to have low value compared to the production of services and this prioritisation appears to be a result of external expectations. There seemed to be a perception of a rift between engaging in RBK and daily tasks.

*Our culture is production*,* I dare say. Slightly scarce resources*,* producing x number of tasks every day*,* that is perhaps also what is appreciated. In a way*,* both internally and externally.* ID1

Even though the managers pointed out many cultural barriers to the use of RBK to develop practice there were also facilitating factors. One of them was creating a professional community at the workplace where they could discuss and work on clinical topics relevant to them and meet external expectations. Having a coordinator in the different departments who had an extra focus on RBK issues, and a network was also seen as important. One of the managers said:

*We don’t sit and google*,* for those who seek something*,* but we find a problem that is with us*,* and then we see if there are any of the people we work with on a regular basis that we can ask*,* don’t you know this? That’s really the way. And we have professional developers who work in each department. We have a quality council every 14 days*,* so we discuss different things*,* issues*,* and try to find solutions to it.* ID11

### Motivational conditions

#### First-line managers perspectives on their own motivation for RBK

Another core theme that were identified was motivational conditions including the managers motivation to seek possible solutions for using RBK. Managers with self-determined motivation for RBK promoted it when seeking answers involving the quality of the services offered to the citizens. One manager said:

*We often get questions on “why and how”. I say to them*,* we are not allowed to answer; just because. We must give a reason for it. That is me. That is printed in me from my nurse education* ID12

Other managers were overwhelmed by the everlasting daily tasks they were obliged to fulfil and even if they were motivated it was challenging for them to find time for RBK. The motivation for RBK withered in the onslaught of daily tasks, hindering its use. The sense of urgency of daily tasks was so high it conquered the slow-paced, time-consuming and long-term based tasks of applying RBK.

*I have to admit*,* the daily tasks occupy more of my time than ideal*,* in relation to the wish one has to sit down and read research. So*,* it ends up being what time you have left in your leisure time.* ID5

According to the managers external obligatory processes such as national controls, where RBK use was expected, were facilitating its use. One manger explained how a control by national supervisory authorities lead to a positive process on improving services through RBK use.

*We are currently preparing for a control; The State Administrators will check use of medicine dispensers and on that matter*,* we try to use research-based knowledge on how it is to improve. … It is a possibility to learn.* ID5

#### First-line managers perspectives on their employee’s motivation for RBK

The managers mentioned that having employees with a master’s degree could be a tool for increased use of RBK as it provides academic competence. Even if this was acknowledged by many of the managers there was a tension between their expectations from employees with a master’s degree, and the motives and intentions of the employees. The managers described their interest as related to developing the services, and solving the daily tasks, while they described the employee’s motivation for a master’s degree to be connected to crossing motives, for example to go from working patient-related, and in shifts, to a day-time job in an office – and not related to a motivation to use RBK.

*They want something different*,* so they take a master’s to either get away from shift work*,* or they take a master’s to change or sharpen their skills to work somewhere else.* ID11

Some managers tried to be strategic and prepare a path for how employees could develop their competence through further education, but they found it challenging due to the employees’ motivation for professional development being closely connected to their private situation. In a life phase with young children and mortgages to pay, the managers experienced that it was considered difficult for the employees to combine a demanding private life with heavy workloads and studies on top. The managers saw this as a common factor that hindered motivation, as employees did not have the necessary energy and made it difficult to acquire the competence needed for RBK.

*There are many paths*,* but we do have some social workers who are particularly interested in mental health work and we have tried and motivated them to take an education*,* but that’s the way it is*,* there are people who are in the establishing phase*,* that’s children*,* and it is difficult*,* even though we almost offer them both study leave and study support*,* it is very difficult.* ID3

### Competence-related conditions

#### Individual competence

The managers highlighted both the competence of the health personnel on an individual level and on a system level. They expressed that a range of competencies were important when applying RBK into practice and that a barrier in municipal health and care services was that they lacked these competencies. These competencies included abilities to know where to search for research literature, how to do a literature search, understanding academic language, critically appraising the information that you find and further contextualizing the information to make it relevant to their clinical practice. Moreover, several also mentioned competencies related to social skills, such as level of maturity in reflection and taking responsibility at work. Having a master’s degree could be a way to achieve these competencies. As one of the managers said:

*Then I can see that having a master’s degree could be very useful in relation to how you acquire it*,* or how to proceed systematically to obtain information […] And where you do not have specialist expertise*,* you must have a greater degree of understanding in relation to how is it that you then assess*,* what is relevant information and how is it that you in a way identify*,* what is it that we do not know in relation to where can we refer or be referred further and correct the question.* ID8

Very few health personnel employed in the departments where we conducted interviews had a master’s degree. The managers expressed that this was a rare competence in the municipalities. More common was having continuing education within a certain topic or field such as wound care or palliative care which provided clinical competences. Several of the managers problematised that it was challenging to find ways to apply RBK from a master’s degree into clinical practice. Maximizing the benefits of knowledge gained through a master’s degree also required that managers possessed the competence to apply this knowledge effectively, including the ability to adapt or reorganise work tasks accordingly. One of the managers said:

*Some of those who have a master’s degree with us work in ordinary clinical work. Perhaps half of them work in ordinary clinical work. And I’m a little unsure about how they use their expertise*,* and what kind of benefit the patients and we derive from it. What I challenge them a bit on is perhaps to contribute to more professional development in the department in relation to hold some internal courses and such*,* what I often experience is that it is I as the top manager who takes the initiative for it.* ID1

#### System for competence enhancement

When it comes to the system level of competence as a barrier of applying RBK into clinical practice, there seemed to be an overload of information, and the managers experienced challenges in navigating within sources of RBK. The managers expressed that there is a jungle of guidelines, reports and research documentation and that it is a challenge to find and prioritise what is relevant for them. Another challenge was to translate this research evidence into a language and actions to support decisions or to make changes in clinical practice. Therefore, having research-based national guidelines made this information more accessible. One of the managers said:

*So*,* I imagine that there could be a small information overload. That’s why it’s good to have very clear guidelines and guides*,* or not always so clear guidelines*,* but at least in a way… if it sounds… Out as laziness*,* that’s it. Here we have the guidelines. This is what we recommend*,* so I think it might be a little more dangerous that there are too many places to get information from.* ID8

Even though there were several barriers for the managers in finding and applying research-based information, there were different support systems that they experienced as useful. One way of achieving competence in the municipalities more systematically was by participating in research projects with external partners from universities, by collaborating with competence centres or participating in networks. Another relevant source of improving competence was webpages and national systems that gather information on clinical procedures, for instance, practical guidance or clinical recommendations. At the same time, the managers pointed out that the research evidence presented in national sources most often focused on very specific and narrow topics, which might not always be relevant to the broader topics in municipal health care. As one manager explained:

*We have “the competence bridge”*,* which is available to all employees*,* and where you can go in and take a lot of courses*,* but also read research and a lot of other work that is done there. It is still very much aimed at the specialist health service. And it may well be that it gets too narrow in a way sometimes because we are much more generalists.* ID1

Several of the managers also talked about possible future facilitators, for instance, in the development of artificial intelligence (AI). They mentioned all the opportunities AI could give to search and summarize information, thus saving time. But they also discussed that it would demand a lot of knowledge about AI and the ability to think critically about what it produces. One of the managers said:

*Of course*,* there is always a risk when it comes to artificial intelligence with shit in*,* shit out*,* but I still think there is a lot of potential in relation to… Let’s say there is a 300-page guide*,* where only maybe 10% is relevant to the particular case you are in. You can then use a digital assistant to say*,* hey*,* I need to extract the most important information from this and that. Can you give me a recommendation based on this guide? At least that’s what I’m hoping for.* ID8

## Discussion

First-line managers within municipal health and care services experience several facilitators and barriers to applying RBK. The core themes identified were connected to motivational, cultural, organisational and competence-related conditions for RBK. Where previous research has mainly focused on the application of RBK within specific professions and through the lens of the health care professionals [6, 25, 27, 44, 45], this study contributes to knowledge on barriers and facilitators within the broader multidisciplinary setting of municipal health and care services. We focus on the first-line managers’ experiences, as we believe that they are either key facilitators or inhibitors of the application of RBK in clinical practise in the municipalities.

The study showed several organisational barriers and facilitators to applying RBK in daily practice in municipal health and care services. The most prevalent barrier mentioned by the managers was the pressure of daily operation of administrating staff and resources. Within their working hours, there was limited time both for the managers and their employees to focus on RBK. In addition, the expectations managers were facing regarding their role in professional development was unclear. According to a systematic review by Birken et al. (2018) mid-level managers have a role in promoting implementation by diffusing information, synthesising information, mediating between strategy and day-to-day activities and selling RBK implementation [46]. In our study, we showed that fulfilling this role is especially challenging in the municipal health and care services compared to hospital care due to limited financial incentives and a lack of personnel expertise and defined roles for application of RBK. It is known from earlier research that organisational elements with clarification of roles and responsibilities, competence specification and information provision are central for fulfilling the potential of professional development [47]. Our study has shown that such organisational elements, demanding resources within the organisation for professional development in general and the use of research in particular, are often lacking at the municipal level.

The different roles of the mid-level managers can be mediated by the implementation climate or the organisational culture for evidence-based practise [46]. The managers in our study highlighted that workplace culture was both a barrier and facilitator in applying RBK in clinical and administrative practice. “Set” cultures and habitual practices, where the employees carry on as they have always done, were seen as a barrier to professional development and applying RBK. However, establishing teams focusing on development within different areas and creating a professional community at the workplace were seen as promoting factors. In municipal health care professionals are in contact with people with varying diagnoses and health challenges, and for a mid-level manager keeping up-to-date on all areas is challenging. Thus, teams that focus on specific areas can function as a support for the managers in implementing RBK. Previous research has shown that having implementation support practitioners or development practitioners, supporting others in adopting and implementing the use of RBK is crucial and necessary, however only if they are integrated into the organisation [48]. Integrating this role into municipal care would be beneficial, however, the results in our study suggest that there are several uncertainties around how to best integrate this role considering funding, responsibility and positioning within the organisation.

First-line managers have a central role in creating a culture and learning community to support the use of RBK [12–14]. In a study by Harvey and colleagues (2019) two leadership approaches towards creating this kind of culture were described, one top-down formalised to ensure adherence to policies and guidelines, and another more enabling and relationship-focused approach to facilitate employees in integrating RBK into a broad evidence-based practice. In the municipalities the mid-level managers tended towards a more relationship-focused approach where they supported employees that showed an interest in RBK. Moreover, the managers represent role models for their employees and personify the organisational culture through demonstrating priorities and setting standards [49]. Six of the managers had either a master’s degree or further education, and this could have been an influential factor in creating cultures working to promote RBK. Another factor influencing the culture for promoting RBK was the variation in professional backgrounds. For instance, several participants stated that within physiotherapy or social work there was a strong focus in their education of keeping up to date on research within their field. This finding is supported by other research showing differences among professions in attitudes and habits towards EBP, suggesting that various professions might have different levels of focus on RBK [50]. However, internally within one profession, the level of knowledge and positive attitudes can vary between settings, leading to the manager taking a more or less active role in implementing EBP [51]. Finally, even if the municipal managers are aiming for a culture or climate for RBK at a system level, their individual attitudes and habits are essential to create a working culture positive towards RBK.

In addition to the collective organisational and cultural challenges for using RBK in municipal health and care services, the results of this study show the importance of personal motivation for RBK among both managers and employees. This motivation encompassed being curious and interested in scientific results and employing the results to seek solutions when having challenging administrative or clinical tasks. However, even though this motivation was evident among some of the managers and employees, the often urgent and overwhelming daily tasks were cornering their attention and drawing their focus away from RBK. This finding is supported by other research showing that even if health care professionals are motivated and competent in using RBK, several organisational barriers have an impact on the ability to increase and maintain the use of RBK at a workplace [52]. In addition to motivation, other personal factors such as self-efficacy in performing professional activities and behaviour have been demonstrated to influence the use of RBK [50]. Our study showed similar results, where the managers highlighted the limited RBK behaviour at their workplace and that only a few of both managers and employees showed an interest in research application. In a recent study from Sweden within a municipal rehabilitation setting, they showed that health care professionals struggled to fulfil their own ambitions on applying RBK and when lacking competence or support to use research they gave up and relied more on experience-based knowledge [45]. The managers in our study confirm these findings, where both their own and their employees’ experiences were the most frequently used knowledge in setting priorities and developing practice. In this context, it is worth recalling that evidence-based practice is not based on experience-based knowledge or RBK alone, but on an integration an integration and interaction between users’ values and preferences, employees’ experiences and skills and RBK [1, 2]. The forms of knowledge represent complementary aspects of knowledge rather than alternatives [31].

Competence, both theoretical and practical, were identified as an overarching theme influencing and influenced by both motivational, cultural and organisational elements. The managers had a central role and impact on all these elements, and they were in a challenging position of fulfilling the expectations of management in the setting of municipal health care. Previous research in municipal care has shown that high and mid-level managers have a central role in supporting implementation processes by having an overview of existing systems and motivating and involving employees [53]. However, several of the first-line managers in our study stated that they were lacking knowledge on research and that this was hindering their application of RBK and their ability to involve employees. Improving competence on RBK among the managers and employees could contribute to a higher degree of RBK use. Educating professional developers in municipal care has been carried out as a strategy in previous research. It has been shown that an educational programme on knowledge translation could improve competence and motivation of development nurses, nevertheless, the organisational culture also needed to fit this way of working [27]. Within management, the leadership and organisational change for implementation (LOCI) strategy, which is a multifaceted implementation strategy to support EBP, has shown to be beneficial in improving implementation leadership and climate [54]. However, this strategy has only been conducted in specialist care within specific clinics focusing on one group of diagnoses, and not in the more complex setting of municipal health care. Another strategy for enhancing competence in municipal care has been assessed in Sweden, where they developed a structure for partnership between care administration in municipal health care and academia [55]. In this study they experienced a transfer of power reducing the academic control, implying that to get this model to succeed the academia might change role from being an administrator and leader to becoming a true collaborator and partner. To conclude, it seems that the organisational structure where RBK is included in strategic work, resources devoted to professional development and ensuring competence in performing RBK activities are all necessary to create a culture and a motivational environment for applying RBK in clinical practice.

This research project stems from an effort to develop a model for implementing the use of research into evidence-based decision-making in municipal health and care services, which has been running for almost three years. The project focuses specifically on infusing and integrating RBK into practical decisions at the service level. In such decisions, the exercise of discretion is necessary. When new research and general academic guidelines and recommendations based on research are applied in new and specific contexts and situations, they must be adapted to local needs and municipal context [56], and professional judgment must be exercised in which the RBK is linked to the experiences of employees and users. Such professional judgment is a prerequisite for individualised treatment and care [57]. It has been important for us to have a clarified understanding of evidence-based practice that is distinct in that such practice involves an integration of systematically obtained RBK, employees’ experience-based knowledge, and the patient’s wishes, needs, and experiences in a given situation. In this understanding, we also rely on an acknowledgement and recognition that practical knowledge, as described by Aristotle, is a multifaceted phenomenon linked to both science and experience-based knowledge [29].

### Strengths and limitations

We should be aware that our data on managers’ understandings and narratives of RBK discourses are not neutral descriptions. As managers, they may have an interest in portraying their department and practices as functioning well and in different ways than ordinary employees would. Collectively, our backgrounds and experience in a broad understanding of evidence-based practice on the municipal level are rather extensive. This was a strength as we had an idea of what to look for and where to look. On the other hand, we went into the interviews with quite a few assumptions and expectations of where the challenges of implementing research-based evidence might be found, as well as what the ‘solutions’ might be. This means that during the interviews, we might have been more interested in asking about and exploring barriers to implementing research-based evidence rather than what kind of research the municipalities can implement and how. Additionally, we had the idea that having more employees with master’s degrees could be part of the solution since that might make people more inclined to consult research as well as better at interpreting that research. Thus, we were a bit surprised during the first few interviews that the experiences presented by the first-line managers were different from what we had imagined. Such surprises loosened up some of our preconceptions and led us to be more open to other interpretations in later interviews, as well as in reading through the transcriptions and analysing and coding the material. Moreover, we interviewed a limited number of managers from a rather limited range of municipalities, which might have had an impact on the level of saturation of the data. Finally, in this study we focused on the perspectives of the first line managers which in municipal health care are often working very close to the clinic and not necessarily involved in strategic planning. Thus, including high-level mangers might have given us even more insight into the contextual factors influencing the use of RBK in municipal health care.

### Implications for clinical practice and research

There are several implications for practice arising from this study. There seems to be a gap between what is expected of the managers in municipal health and care services, both taking charge of the organisational and cultural aspects as well as ensuring the right competence and motivation of the employees, and what they can do. There is a need for support on different levels, both nationally and locally, to enable the application of RBK. On the national level, gathering research-based information on a limited number of platforms and in a format that applies to clinical and administrative practices would promote the use of RBK. Another promoter on the national or regional level would be having networks or centres to support the application of RBK. On the local level, ensuring that RBK has a place in strategic documents, ensuring strategic planning of competence for RBK, having a focus on RBK activities and having employees with the role of professional developers supporting the managers in the application of RBK could be relevant measures.

This study also points to several implications for future research. While our focus has been on first-line managers, it would be valuable to explore how both employees and higher-level managers perceive the application of RBK within municipal health and care services. Further research could also examine how RBK is positioned relative to user-based and experience-based knowledge in these settings. In addition, investigating effective strategies for overcoming barriers to the implementation and integration of RBK in practice would be an important area for continued inquiry.

## Conclusion

According to our informants, the most significant barrier to promoting the use of research in everyday municipal health and care practice is the constant pressure of routine clinical and administrative tasks on both managers and employees. Changing entrenched practices and established work cultures also poses a challenge. First-line managers described the establishment of change-oriented teams and the creation of professional learning communities as important facilitators. Managers play a central role in fostering these learning environments. Their own motivation—and their perceptions of their employees’ motivation—is crucial. Motivation involved curiosity and an interest in scientific findings, as well as a willingness to apply these findings to address complex clinical and administrative challenges. However, the urgency and volume of daily tasks often divert attention away from RBK. Competence intersects with these issues, shaping and being shaped by motivational, cultural, and organisational factors.

Finally, there also appears to be a gap between the expectations placed on managers within municipal health and care services and what they are realistically able to accomplish. Support is needed at multiple levels, both national and local. It is essential in such efforts to recognise that evidence and knowledge are multifaceted phenomena, drawing not only on science but also on experience-based insights within concrete practice situations.

## Electronic supplementary material

Below is the link to the electronic supplementary material.

Supplementary material 1

## Data Availability

Interview transcriptions generated and analysed during the current study are not publicly available since the small sample might lead to identification of the participants but are available from the corresponding author on reasonable request.
