# Supplementary material for: Barriers and facilitators for using research-based knowledge - A qualitative study on first-line managers’ perspectives in municipal health and care services
Source: BMC Health Serv Res. 2026 May 7;26:892. doi: 10.1186/s12913-026-14666-0 (PMC13321641; doi:10.1186/s12913-026-14666-0)
Supplement: Supplementary file 1 — Supplementary material 1 [file 12913_2026_14666_MOESM1_ESM.docx]

**Appendix 1: Interview guide**

Barriers and facilitators for using research-based knowledge. A qualitative study on first-line managers' perspectives in municipal health and care services

The research question underlying the study was: What are the barriers and facilitators for the use of research-based knowledge in municipal health and care services?

Initiate by explaining the purpose of the interview and themes in the interviews.

Can you start by telling us a little about the main content of your position?

Application of research-based knowledge:

- Can you reflect on using research that is published in the work of your department? How relevant is it? How important is it? To what extent is it done?
- How do you go about finding/identifying relevant research? How easy/difficult is it? Can you give some examples?
- How do you apply/use research? Can you give some examples? Any good stories about this?
- Have you succeeded in applying research-based knowledge? Why do you think you succeeded? Have you experienced not succeeding? Please elaborate.
- Can you reflect on: What hinders use? What promotes use? Is there something in the work environment/culture that hinders or promotes this?
- Do you experience any contradictions/conflicts between daily operations and spending time on applying research-based knowledge?
- To what extent do employees have time to familiarize themselves with relevant research? Please distinguish between different types of positions.

Is there anything else that you would like to elaborate on?
